# Assessing scale‐dependent effects on Forest biomass productivity based on machine learning

**DOI:** 10.1002/ece3.9110

**Published:** 2022-07-13

**Authors:** Jingyuan He, Chunyu Fan, Yan Geng, Chunyu Zhang, Xiuhai Zhao, Klaus von Gadow

**Affiliations:** ^1^ Research Center of Forest Management Engineering of State Forestry Administration Beijing Forestry University Beijing China; ^2^ Faculty of Forestry and Forest Ecology Georg‐August‐University Göttingen Germany; ^3^ Department of Forest and Wood Science University of Stellenbosch Matieland South Africa

**Keywords:** above‐ground biomass, productivity, random Forest algorithm, random spatial sampling, scale dependence

## Abstract

Estimating forest above‐ground biomass (AGB) productivity constitutes one of the most fundamental topics in forest ecological research. Based on a 30‐ha permanent field plot in Northeastern China, we modeled AGB productivity as output, and topography, species diversity, stand structure, and a stand density variable as input across a series of area scales using the *Random Forest (RF)* algorithm. As the grain size increased from 10 to 200 m, we found that the relative importance of explanatory variables that drove the variation of biomass productivity varied a lot, and the model accuracy was gradually improved. The minimum sampling area for biomass productivity modeling in this region was 140 × 140 m. Our study shows that the relationship of topography, species diversity, stand structure, and stand density variables with biomass productivity modeled using the RF algorithm changes when moving from scales typical of forest surveys (10 m) to larger scales (200 m) within a controlled methodology. These results should be of considerable interest to scientists concerned with forest assessment.

## Graphical abstract

Based on a 30‐ha permanent field plot in Northeastern China, we modeled AGB productivity as output, and topography, species diversity, stand structure, and a stand density variable as input across a series of area scales using the Random Forest algorithm. As the grain size increased from 10m to 200m, we found that the relative importance of explanatory variables that drove the variation of biomass productivity varied a lot, and the model accuracy was gradually improved. The minimum sampling area for biomass productivity modeling in this region was 140 m × 140 m. These results should be of considerable interest to scientists concerned with forest assessment.

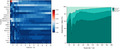

## 1 INTRODUCTION

Terrestrial ecosystems play an important role in regulating the global and local climates (Gadow et al., 2021; Hwan & Chun, 2011; Zhou et al., 2013), and carbon cycles (Kuribayashi et al., 2017; Zhao et al., 2012), and maintaining biodiversity (Kitayama et al., 2018; Ren et al., 2017). Among these terrestrial ecosystems, the forest ecosystem is the largest C reservoir, which comprises more than 80% and 40% of the global terrestrial C pools above‐ground and below‐ground, respectively (Dixon et al., 1994; Luo et al., 2020; Pan et al., 2011). Tree trunks and branches contain a massive ratio of these C, which is called above‐ground biomass (AGB) (Fahey et al., 2010; Fotis et al., 2018). The relationships between biodiversity and AGB or productivity have drawn ample attention in ecology and conservation biology (Cadotte, 2015; Cardinale et al., 2012; Isbell et al., 2011; Liang et al., 2016; Qiao et al., 2021). Numerous debates have been initiated with respect to the impacts of species diversity on productivity or AGB, with positive, negative, hump‐shaped or U‐shaped relationships (Mittelbach, 2010; Poorter et al., 2017; Ruiz‐Benito et al., 2014; Whittaker, 2010; Zhang et al., 2015). Most studies have attended to scrutinize biomass instead of productivity (Holdaway et al., 2016). However, strictly speaking, the concepts of them are different in forest ecosystems (Chisholm et al., 2013; Schmid et al., 2009), though they are sometimes correlated positively (Stegen et al., 2011). Thus, biomass and productivity should be analyzed in different ways (Chisholm et al., 2013). The ambiguous results above declare our limited cognition on the diversity and productivity. Estimating forest biomass per unit area is important and challenging, and key to estimating forest carbon stock. Net biomass change per unit of area and time is an important index to measure forest productivity and effects of management (Hao et al., 2018; Luo et al., 2019).

Stand structure factors, such as stem density, tree size variation, and stand structural diversity, are critical components in characterizing forest productivity (Ali et al., 2016, 2019a; Morin et al., 2011; Rodriguez‐Hernandez et al., 2021; Sullivan et al., 2017; Yachi & Loreau, 2007). It has been demonstrated that various layered stand structures imply multiple canopy, which contributes to more capture of light and other resources (Yachi & Loreau, 2007). Environmental conditions (i.e., topography heterogeneity in this study) in elevation, aspect and slope impact nutrient, plant traits, water availability, and biodiversity patterns directly and indirectly, which shapes them decisive predictors to project productivity (de Castilho et al., 2006; Liu et al., 2014; Zhang et al., 2012, 2016). Tree biomass has strong links to stand density (Dahlhausen et al., 2017; Mejstřík et al., 2022; Wertz et al., 2020; Xue et al., 2012). Węgiel et al. (2018) revealed that higher stand density might lead to higher total biomass production and carbon stock. The associations across diversified abiotic and biotic determinants of productivity remain debated (Ali et al., 2019a,b; Rodriguez‐Hernandez et al., 2021). Moreover, the character of scale in unraveling the relationships is less well‐known (but see Rodriguez‐Hernandez et al., 2021). Community often shows large spatial variability due to environmental, community process, and disturbing factors (Chave et al., 2003; Mascaro et al., 2011). Generally, greater plot sizes reflect the characteristics of the community more accurately, but this ideal cannot be achieved because of the constraints of available funding. Therefore, it is necessary to design a suitable plot size to meet both scientific requirements and cost savings (Bradford et al., 2010; Chave et al., 2003; Kral et al., 2010; Laumonier et al., 2010; Pyle et al., 2008; Wagner et al., 2010). It is important to determine the smallest area of sampling plot that can be representative of the characteristics of the whole community (e.g., species composition and structure) (Peng & Guo, 2016). For instance, the sample plot area in most studies is generally smaller than 1 ha and they tend to focus on a single grain size (Holdaway et al., 2016; Li et al., 2019; Sande et al., 2017; Xu et al., 2015; Yuan, Ali, et al., 2018), which might lead to different results when viewed at even smaller or larger spatial extents. Can the survey results based on the quadrats of the above area truly reflect forest productivity? Is there a minimum area that can fully reflect the spatial variability of stand productivity? So far, there seems to be no reliable evaluation basis. Furthermore, several studies have used the species–area curve to determine the minimum plot area (Harte et al., 2009; Kallimanis et al., 2008; Tikkanen et al., 2009), but this cannot reflect the characteristics of population and community structure. Thus, it is imperative and imminent that appraising the impact of biotic and abiotic factors on productivity at different spatial scales.

Although forest growth is a complex nonlinear continuous process, big data and machine learning algorithm provide new possibilities for productivity modeling. The Random Forest (RF) algorithm has been used to deal with the relationships between explanatory and response variables without assuming specific mathematical equations and statistical assumptions (De'ath, 2007). It can effectively avoid over‐fitting, evaluate the relative importance of explanatory variables, and is insensitive to collinearity among explanatory variables (Prasad et al., 2006) when facing too many data dimensions. Therefore, we used RF in our study, which can better help us decide on the appropriate sampling sizes.

China's forest ecosystems represent an important carbon sink, especially those in northeastern China. This study is based on a 30‐ha (500 × 600 m) monitoring field plot established in an old‐growth broadleaved Korean pine forest in Jiaohe Forest Experimental Administration of Jilin Province. This large plot allows the study of the spatial scale effects on productivity modeling. We used RF algorithm to simulate biomass productivity. Topography factors, diversity factors, stand structure factors, and a density factor were selected as explanatory variables, while biomass productivity was selected as response variable. The following questions were addressed in this study: (1) What is the minimum plot area for predicting the biomass productivity with relatively high accuracy? By analyzing the prediction accuracy of the RF models built at different scales, we may suggest a minimum sampling area for productivity. (2) What are the similarities and differences in variables importance across different grain scales? This study is thus expected to provide new insights regarding the estimation of biomass productivity in a natural forest.

## 2 MATERIALS AND METHODS

### 2.1 Study area

**FIGURE 1:**
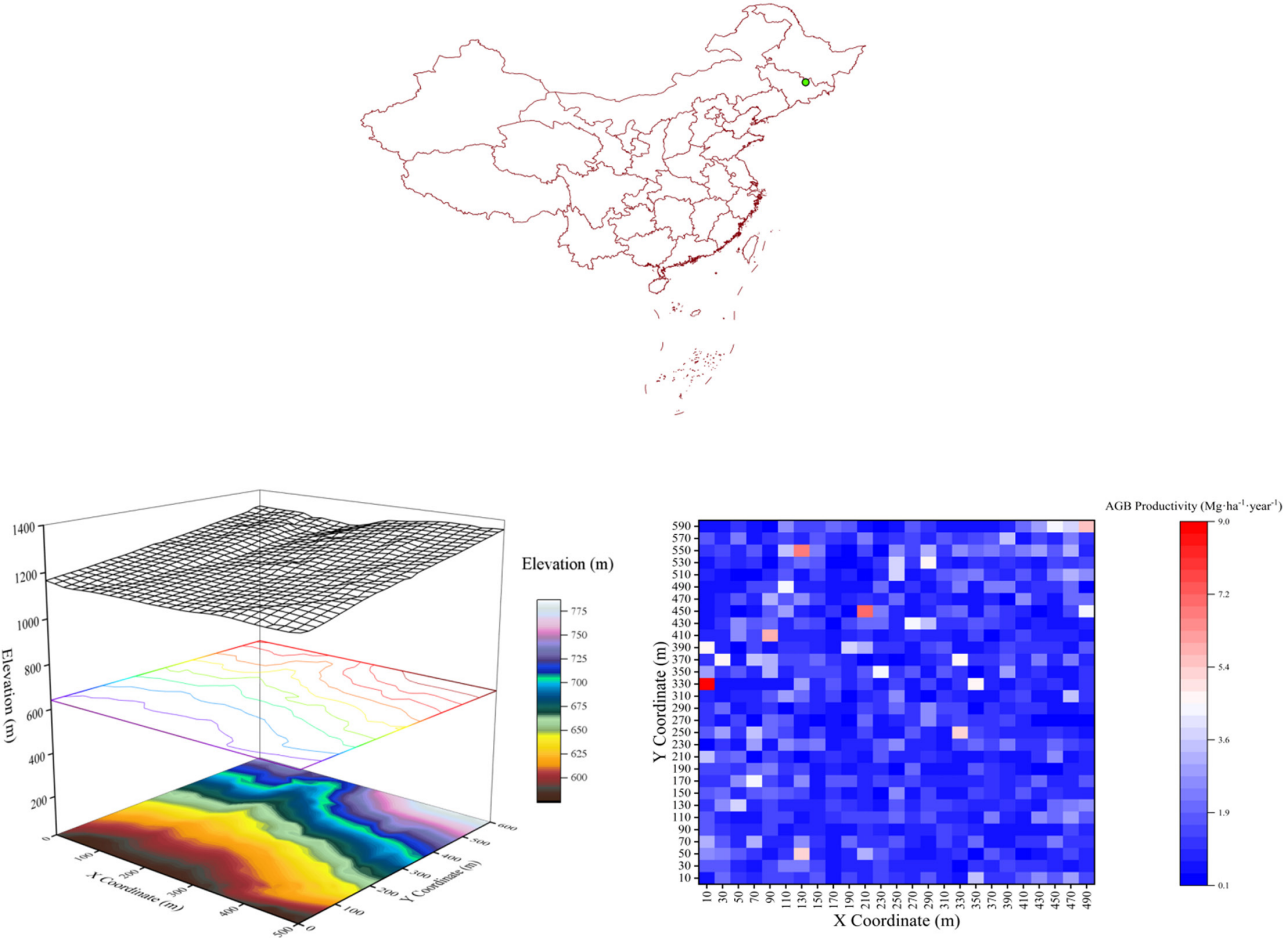
Top: Location of the study area in northeastern China. Bottom‐left: Map depicting elevation patterns. The color from dark to light means the observed values are from low to high. Bottom‐right: Map depicting forest biomass productivity patterns at the scale of 20 m. The color from blue to red means the observed values are from low to high

The study site is located in the Jiaohe Management Bureau of the Forest Experimental Administration in Jilin Province, China, in a temperate continental monsoon climate with a mean annual temperature of 3.8°C and mean annual precipitation of 695.9 mm. The average temperature of the hottest month is 21.7°C in July, and the average temperature of the coldest month is −18.6°C in January (Xu et al., 2019). The soil type is a dark brown forest soil, with an average depth of 45 cm. A permanent observational field plot, covering an area of 30‐ha (500 m × 600 m), was established in 2010 at 43°57.928′–43°58.214’N, 127°45.287′–127°45.790′E. The elevation ranges between 576 m and 784 m. The forest is a typical unmanaged coniferous and broadleaved‐mixed forest, which has been left undisturbed for more than 50 years (Figure 1). The dominant species are *Pinus koraiensis*, *Tilia amurense*, *Ulmus laciniata*, *Acer mono* and *Betula costata*. We completely surveyed the plot in two consecutive inventories: the first inventory was conducted in 2010 and the second in 2015. All of the woody plants with diameters at breast height (DBH) ≥ 1 cm were tagged and mapped, and their species were identified. The tree DBHs, heights, crown widths, and height‐to‐live crowns were measured in both censuses.

### 2.2 Sample design and dataset

A random spatial sampling design was employed to investigate the relationship between forest biomass productivity and topographic, species diversity, stand structure, and a stand density variable at different spatial scales. For our design, different spatial scales represented a series of square‐shaped quadrats of different size, which increased from 10 × 10 m to 200 × 200 m in stepwise 5 m increase of the side length. Thus, a total of 39 spatial scales ranging from 10 to 200 m were studied: 10 × 10 m, 15 × 15 m, …, 195 × 195 m, and 200 × 200 m. For each of the 39 quadrat sizes, 100 quadrats of the same size were randomly placed in the permanent observational field plot. We set up a buffer zone in the plot that extended from the boundary of the plot to half the side length of the quadrat. The center of the quadrat was extracted from the unbuffered zone to ensure that all the quadrats are in the plot. For each quadrat, tree species, DBH, and heights were extracted.

Four quadrat‐level biotic and abiotic attributes were derived: (a) topographic variables (Topography), (b) species diversity variables (Diversity), (c) stand structure variables (Structure), and (d) stand density variable (Density). In our study, the quadrat biomass productivity (*P*) as output was modeled using the RF algorithm with the mentioned variables above as inputs. Thus, the basic model form was expressed as follows:

(1) $$P=f\left(\text{Topography},\text{Diversity},\text{Structure},\text{Density}\right)$$

For each of the 39 scales, we utilized a completely randomized spatial sampling design, which was similar to Luo et al. (2019). Because of each quadrat standing for a virtual sample plot, the sampling design facilitated the study between biomass productivity and four biotic and abiotic attributes along the area scales. The 100 quadrats of the same size were randomly placed in the study area with replacement. This process was repeated 1000 times.

### 2.3 Biomass productivity

In our study, the AGB of all individual trees were estimated using a set of region‐specific allometric equations with DBH as an independent variable (Table A.2) (Yuan, Wang, et al., 2018). Each quadrat's total AGB was computed as the sum of the AGB of all the individuals within the quadrat. According to Prado‐Junior et al. (2016) and Luo et al. (2019), forest productivity (*P*) of the quadrats could be measured as the periodic annual increment of total AGB per unit area and time. Only individuals with a DBH ≥5 cm were included since such trees were responsible for most of the AGB (Chiang et al., 2016; Hao et al., 2018).

### 2.4 Topographic variables

The plot was subdivided into 750 cells of 20 × 20 m, and the elevation of four corners of each cell was measured during plot establishment. Ordinary kriging was employed to construct a trend surface (Luo et al., 2019). Based on the trend surface, we extracted the elevation values of the center and four corners of each quadrat which we randomly placed in the permanent observational field plot. We further estimated slope and aspect values (Luo et al., 2019). When measuring the slope, any three of the four corners of the quadrat would form a plane, and the average angle between the plane and the horizontal plane was the slope of the quadrat. The average value of the angle between the plane composed of the corners and the vertical plane of the quadrat is the aspect value of the quadrat. We selected elevation (*E*), slope (*SLO*) and aspect (*ASP*), that is, *CE*, *SLC* as topographic variables, following Xiang et al. (2016):

(2) $${\mathrm{CE}}_{\mathrm{ij}}=\cos\left({\mathrm{ASP}}_{\mathrm{ij}}\right)\times \ln\left(E_{\mathrm{ij}}\right)$$

(3) $$S{\mathrm{LC}}_{\mathrm{ij}}=\tan\left({\mathrm{SLO}}_{\mathrm{ij}}\right)\times \cos\left({\mathrm{ASP}}_{\mathrm{ij}}\right)$$

### 2.5 Diversity variables

Seven indices were used to quantify the diversity of the species. Species richness (*Richness*) refers to the number of tree species that were present in each quadrat. We also studied Hill numbers (Hill, 1973) and species evenness as diversity measures. Hill number is defined as follows:

(4) Dqij=∑ijk=1Richnessijpijkq11−q

where ijk means kth species of the ijth quadrat, $p_{\mathrm{ijk}}$ is the proportion of the kth species in the ijth quadrat in terms of stem number, q is the sensitivity of the measure to the relative frequencies.

As q tends to 1, Hill number is the exponential of the Shannon entropy (Jost, 2006). As q = 2, Hill number is equivalent of the Gini‐Simpson diversity, the inverse of the Gini‐Simpson index (Jost, 2006) as follows: In addition, we also calculated species evenness (Pielou, 1975) as diversity measures.

(5) D1ij=exp−∑ijk=1Richnessijpijklnpijk

(6) D2ij=∑ijk=1Richnessijpijk2−1

(7) E1ij=D1ijlnS

(8) E2ij=D2ijlnS

Additionally, we considered the abundance‐based coverage estimator (*ACE* index) (Anne & Mark, 1993) and the *Chao1* index (Anne, 1984) as diversity variables as well because *ACE* index can be used to estimate the number of species not yet observed in the community and *Chao1* index can be used to estimate the total number of species and is sensitive to rare species. The greater the *ACE* value, the more is the real species in the community. The greater the *Chao1* value is, the greater is the species richness.

(9) $${\mathrm{ACE}}_{\mathrm{ij}}=S_{{\text{abund}}_{\mathrm{ij}}}+\frac{S_{{\text{rare}}_{\mathrm{ij}}}}{C_{{\mathrm{ace}}_{\mathrm{ij}}}}+\frac{F_{1_{\mathrm{ij}}}}{C_{{\mathrm{ace}}_{\mathrm{ij}}}}\gamma_{{\mathrm{ace}}_{\mathrm{ij}}}^{2}$$

(10) $$\gamma_{{\mathrm{ace}}_{\mathrm{ij}}}^{2}=\max\left[\frac{S_{{\text{rare}}_{\mathrm{ij}}}}{C_{{\mathrm{ace}}_{\mathrm{ij}}}}\frac{\sum_{\mathrm{ijk}=1}^{10}i\left(i-1\right)F_{1_{\mathrm{ij}}}}{N_{{\text{rare}}_{\mathrm{ij}}}\left(N_{{\text{rare}}_{\mathrm{ij}}}-1\right)}-1,0\right]$$

(11) $$N_{{\text{rare}}_{\mathrm{ij}}}=\sum_{\mathrm{ijk}=1}^{10}{\text{ijkF}}_{\mathrm{ijk}}$$

(12) $$C_{{\mathrm{ace}}_{\mathrm{ij}}}=1-\frac{F_{1_{\mathrm{ij}}}}{N_{{\text{rare}}_{\mathrm{ij}}}}$$

where $S_{\text{abun}d_{\mathrm{ij}}}$ is the number of abundant (abundance threshold > *n*) species in the ijth quadrat; $S_{\mathrm{rar}e_{\mathrm{ij}}}$ is the number of rare (abundance threshold ≤ *n*) species in the ijth quadrat; $F_{1_{\mathrm{ij}}}$ refers to the number of species represented by one individual only in ijth quadrat; $\gamma_{\mathrm{ac}e_{\mathrm{ij}}}^{2}$ represents the estimated coefficient of variation of rare species in the ijth quadrat. *n* = 10 is commonly used as the abundance threshold to classify abundant or rare species. At this point, $\gamma_{\mathrm{ac}e_{\mathrm{ij}}}^{2}$, $N_{\mathrm{rar}e_{\mathrm{ij}}},$ and $C_{\mathrm{ac}e_{\mathrm{ij}}}$ can be calculated by the above formula, where $F_{\mathrm{ijk}}$ is the number of the ijkth species represented by individuals.

(13) $$\text{Chao}1_{\mathrm{ij}}={\text{Richness}}_{\mathrm{ij}}+\frac{F_{1_{\mathrm{ij}}}\left(F_{1_{\mathrm{ij}}}-1\right)}{2\left(F_{2_{\mathrm{ij}}}+1\right)}$$

where $F_{2_{\mathrm{ij}}}$ refers to the number of species which contain at least two individuals in ijth quadrat.

### 2.6 Stand structural variables

Our stand structural variables include the number of stems (*Nall*), the number of large trees (*N60*, i.e., number of stems with DBH ≥60 cm), skewness of the log‐normal distribution fitted to all individuals' DBH data (*skewness*), the shape parameter of the Weibull distribution fitted to the same data (*shape*), Shannon index of DBH (*DBHShannon*), Simpson index of DBH (*DBHSimpson*), gini index of DBH (*GiDBH*), coefficient of variation of DBH (*CVDBH*), Shannon index of tree height (*HShannon*), Simpson index of tree height (*HSimpson*), gini index of tree height (*GiH*) and coefficient of variation of tree height (*CVH*). The calculation method of a structural diversity index is as follows: Taking 2 cm as a diameter class width and 1 m as a tree height class width, the number of DBH classes or tree height classes, and the number of individuals in each class in each quadrat were calculated respectively. According to the calculation, formulas of Shannon index and Simpson index, *DBHShannon*, *DBHSimpson*, *HShannon*, and *HSimpson* were calculated by substituting the number of species with the number of DBH classes or tree height classes respectively:

(14) $${\text{GiDBH}}_{\mathrm{ij}}=\frac{\sum_{\mathrm{ijm}=2}^{N_{\mathrm{ij}}}\left(2k-N_{\mathrm{ij}}-1\right){\mathrm{BA}}_{\mathrm{ijm}}}{\sum_{\mathrm{ijm}=2}^{N_{\mathrm{ij}}}\left(N_{\mathrm{ij}}-1\right){\mathrm{BA}}_{\mathrm{ijm}}}$$

(15) $${\mathrm{GiH}}_{\mathrm{ij}}=\frac{\sum_{\mathrm{ijm}=2}^{N_{\mathrm{ij}}}\left(2k-N_{\mathrm{ij}}-1\right)H_{\mathrm{ijm}}}{\sum_{\mathrm{ijm}=2}^{N_{\mathrm{ij}}}\left(N_{\mathrm{ij}}-1\right)H_{\mathrm{ijm}}}$$

(16) $${\text{CVDBH}}_{\mathrm{ij}}=100\%\frac{\sqrt{\frac{1}{N_{\mathrm{ij}}}{\left({\mathrm{DBH}}_{\mathrm{ijm}}-\mu_{\mathrm{ij}}\right)}^{2}}}{\mu_{\mathrm{ij}}}$$

(17) $${\mathrm{CVH}}_{\mathrm{ij}}=100\%\frac{\sqrt{\frac{1}{N_{\mathrm{ij}}}{\left(H_{\mathrm{ijm}}-\lambda_{\mathrm{ij}}\right)}^{2}}}{\lambda_{\mathrm{ij}}}$$

where $\mathrm{DB}H_{\mathrm{ijm}}$ is the diameter at breast height of the mth individual in the ijth quadrat; $\mu_{\mathrm{ij}}$ is the average DBH of all individuals in the ijth quadrat; $H_{\mathrm{ijm}}$ is the tree height of the mth individual in the ijth quadrat; $\lambda_{\mathrm{ij}}$ is the average tree height of all individuals in the ijth quadrat.

### 2.7 Density variables

We calculated the Reineke Stand Density Index (*SDI*) for each quadrat.

(18) $${\mathrm{SDI}}_{\mathrm{ij}}=N_{\mathrm{ij}}\times {\left(\frac{20}{D_{g_{\mathrm{ij}}}}\right)}^{\beta }$$

where $D_{g_{\mathrm{ij}}}$ is the mean diameter in the ijth quadrat, and β is the allometric exponent that expresses the relation between tree size and number of trees.

Definition, units for the key forest attributes studied here are summarized in Table A.3.

### 2.8 “Random Forest” algorithm

The RF is a popular machine learning algorithm based on multiple decision trees (Leo, 2001). It can deal with both regression problems and classification problems. In the process of splitting each child node, some variables from all candidates for the splitting variables are selected randomly, and then the optimal ones are determined (Wang & Wang, 2021). For the initial dataset, the training data are chosen randomly to build the model, the data not included are defined as “out‐of‐bag” (OOB) (Catani et al., 2013). The RF error is approximated by the OOB error during the training process (Naghibi et al., 2017).

A total of 22 explanatory variables including 2 topographic variables, 7 species diversity variables, 12 stand structural variables, and 1 stand density variable were selected in this study to participate in the modeling of biomass productivity, resulting in a very large data dimension. Therefore, to avoid the “dimensionality curse” and to reduce the time required for RF algorithm modeling, it is essential to screen the predictors before modeling (Chandrashekar & Sahin, 2014; Zarshenas & Suzuki, 2016). A novel feature selection method, the Boruta algorithm (Kursa & Rudnicki, 2010), was adopted in this study. The goal is to screen all sets of features associated with the response variable, rather than selecting for a specific model the set of features that minimizes the model cost function, and thus to screen for explanatory variables (Kursa & Rudnicki, 2010) more comprehensively and efficiently. The Boruta algorithm has been applied in geology (Pourghasemi et al., 2020), hydrology (Amiri et al., 2019), and ecology (Arjasakusuma et al., 2020; Dobrowolska & Bolibok, 2019; Poona et al., 2016) studies.

Hyper‐parameter tuning is a tedious but crucial task for machine learning algorithms (Ou et al., 2019), which aims to improve model fitting and the reduction of prediction errors. In the RF model, the hyper‐parameter “mtry” specifies the number of variables in the node for a binary tree, with a default value of one‐third of the number of dataset variables (Leo, 2001). The “tuneRF” function from the “randomForest” package in the R software was employed to determine the optimal “mtry,” at which point the corresponding OOB error is the smallest. The hyper‐parameter “ntree,” with a default value of 500, controls the number of decision trees (Leo, 2001). However, “ntree” values ranged from positive integers, and it is not feasible to train models with corresponding all “ntree” values and evaluate them. The common practice to solve this problem is to select a certain value range and step size for the hyper‐parameters and evaluate the model trained by the candidate hyper‐parameters to screen the optimal hyper‐parameters. This compromise method of determining hyper‐parameters balances computational costs and model performance, making the process of machine learning feasible (Ou et al., 2019).

Therefore, in this study, the range of “ntree” values was set at [0, 7000]. The function “randomForest” was used to calculate the model error corresponding to each “ntree” value under the optimal “mtry” values. The function “plot” was used to plot the model error versus the number of decision trees, showing that the error decreased gradually as the decision tree increased, and finally leveled off, at which point the number of decision trees, that is, the optimal value of “ntree,” was taken. Stationarity in this study was defined as follows: Within the value range of “ntree,” the step length was set to 500. The difference in a corresponding error of the ith candidate value and the (i + 1) th candidate value was calculated. If the difference was less than 0.1, the “stationarity” was reached. The ith candidate value was determined as the value of the optimal “ntree.” If the difference was greater than 0.1 across the range of “ntree” values, the threshold was upregulated by 0.05, until the optimal “ntree” value was taken. We conducted model selection and estimation of hyper‐parameters using the package “randomForest” of R version 4.0.4 (R Core Team, 2021).

We first randomly selected 70% of the data for training and 30% for testing. Common criteria for assessing the goodness‐of‐fit of model predictions have historically been the mean error (ME), the mean absolute error (MAE), the mean relative error (MRE), the root‐mean‐squared error (RMSE), the relative RMSE (rRMSE), the coefficient of determination (*R* ^2^). Lower absolute ME and absolute MAE values indicate higher goodness‐of‐fit. Lower MRE, RMSE, and rRMSE values indicate higher goodness‐of‐fit. Higher *R* ^2^ values indicate higher goodness‐of‐fit.

(19) $$\mathrm{ME}=\frac{\sum_{p=1}^{n}\left({\mathrm{obs}}_{p}-{\mathrm{est}}_{p}\right)}{n}$$

(20) $$\mathrm{MAE}=\frac{\sum_{p=1}^{n}\left|{\mathrm{obs}}_{p}-{\mathrm{est}}_{p}\right|}{n}$$

(21) $$\mathrm{MRE}=\frac{\sum_{p=1}^{n}\left({\mathrm{obs}}_{p}-{\mathrm{est}}_{p}\right)}{\sum_{p=1}^{n}{\mathrm{obs}}_{p}}$$

(22) $$\text{RMSE}=\sqrt{\frac{\sum_{p=1}^{n}{\left({\mathrm{obs}}_{p}-{\mathrm{est}}_{p}\right)}^{2}}{n}}$$

(23) $$\text{rRMSE}=\frac{\sqrt{\frac{\sum_{p=1}^{n}{\left({\mathrm{obs}}_{p}-{\mathrm{est}}_{p}\right)}^{2}}{n}}}{\frac{\sum_{p=1}^{n}{\mathrm{obs}}_{p}}{n}}\times 100$$

(24) $$R^{2}=1-\frac{\sum_{p=1}^{n}\left({\mathrm{obs}}_{p}-{\mathrm{est}}_{p}\right)}{\sum_{p=1}^{n}\left({\mathrm{obs}}_{p}-\frac{\sum_{p=1}^{n}{\mathrm{obs}}_{p}}{n}\right)}$$

where $\mathrm{ob}s_{p}$ and $\mathrm{es}t_{p}$ are the p_th_ observation and estimate, respectively, and n is the number of observations.

## 3 RESULTS

### 3.1 Evaluating the RF simulation accuracy at different area scales

**FIGURE 2:**
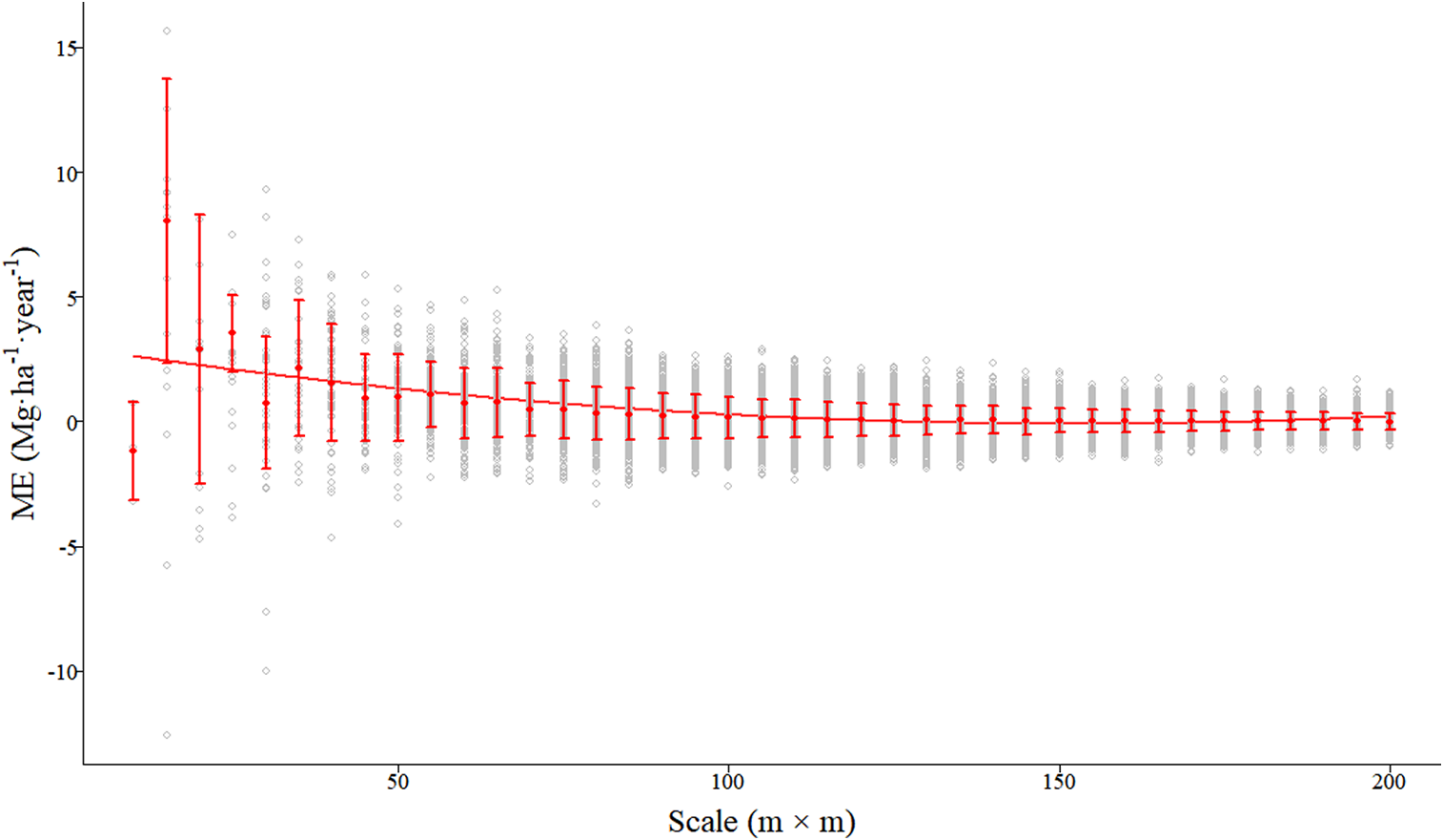
Boxplot of the mean error (ME) changes modeled by the RF algorithm at each quadrat area scale. The solid line represents the mean trend line values, whereas dots with horizontal bars represent mean the ME for each quadrat size value and its standard deviation (SD)

**FIGURE 3:**
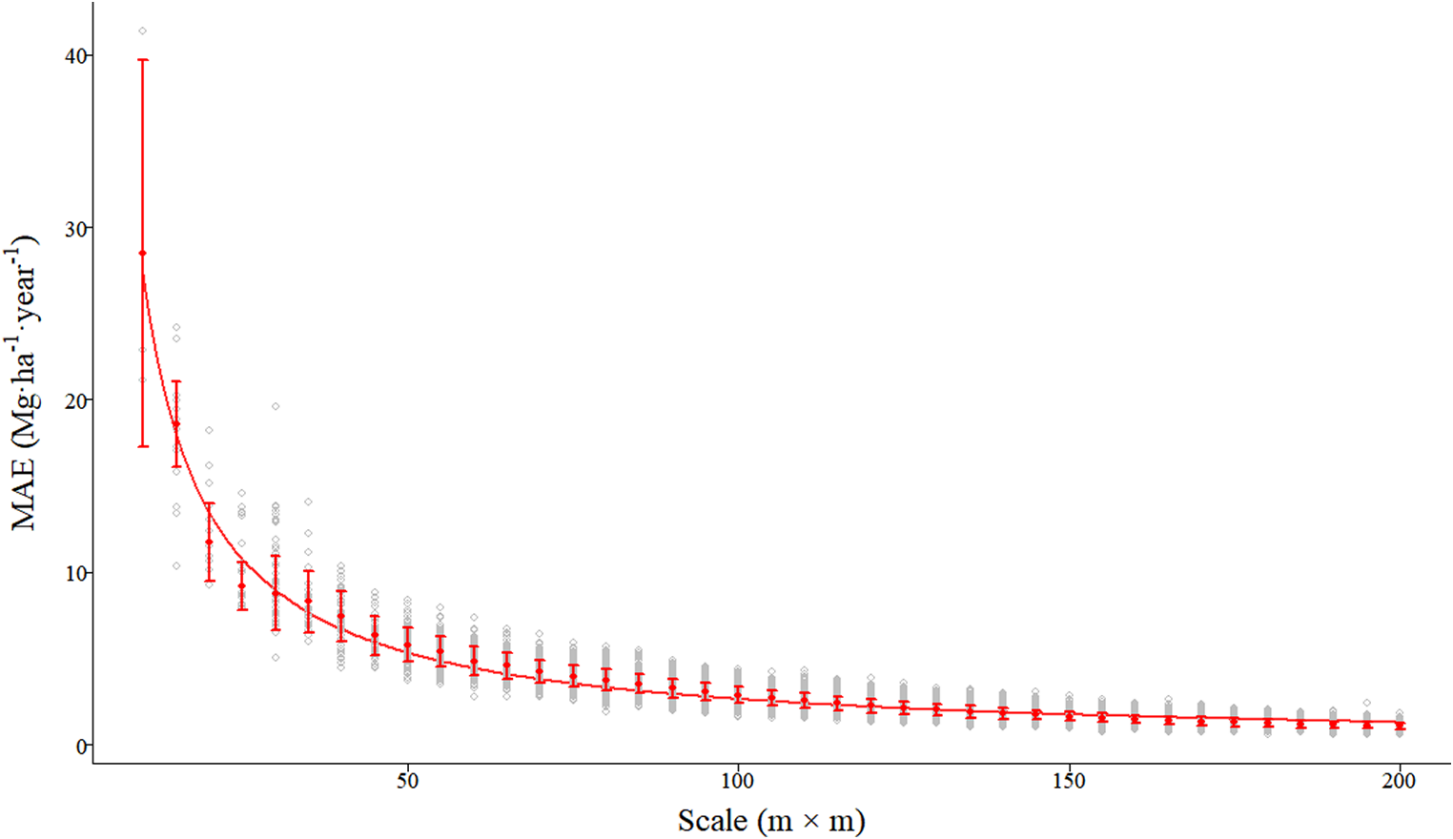
Boxplot of the mean absolute error (MAE) changes modeled by the RF algorithm at each quadrat area scale. The solid line represents the mean trend line values, whereas dots with horizontal bars represent mean the MAE for each quadrat size value and its standard deviation (SD)

**FIGURE 4:**
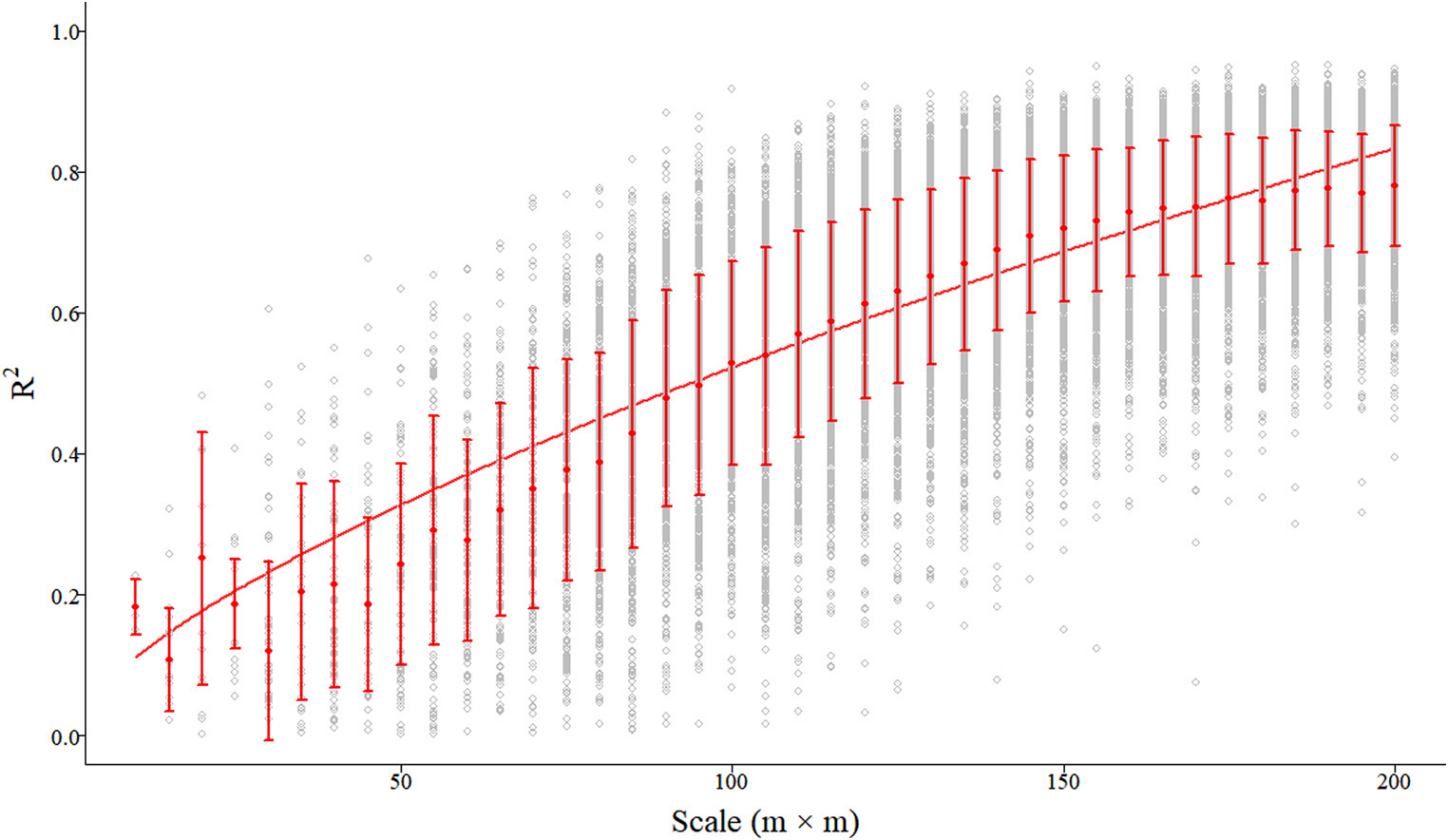
Boxplot of the coefficient of determination (*R* ^2^) changes modeled by the RF algorithm at each quadrat area scale. The solid line represents the mean trend line values, whereas dots with horizontal bars represent mean the *R* ^2^ for each quadrat size value and its standard deviation (SD)

For the test data, ME and MRE showed the same pattern with smaller fluctuating ranges and achieved maximum of median absolute values at the scale of 15 m (9.2157 Mg·ha^−1^·year^−1^ and 0.2932, respectively), and minimum of median absolute values at the scale of 185 m (0.0019 Mg·ha^−1^·year^−1^ and 0.0001, respectively; Figure 2; Figure A.1) with increasing quadrat area. MAE had the highest median value (28.4703 Mg·ha^−1^·year^−1^) at 10 m and had the lowest median value at 200 m (1.0645 Mg·ha^−1^·year^−1^; Figure 3). RMSE continually decreased at all scales and resulted in the highest and lowest median values (29.7232 Mg·ha^−1^·year^−1^ and 1.3658 Mg·ha^−1^·year^−1^) at 10 and 200 m, respectively (Figure A.2). *R* ^2^ had an increasing trend with increasing quadrat area scales, reaching the highest median values at the scale of 200 m (0.79; Figure 4). The rRMSE decreased with increasing scale, and the lowest median value (9.49%) were at the 200 m plot size (Figure A.3). Thus, the optimal scale for estimating forest biomass productivity in this region may be chosen based estimated accuracy.

### 3.2 Importance of explanatory variables at different scales

**FIGURE 5:**
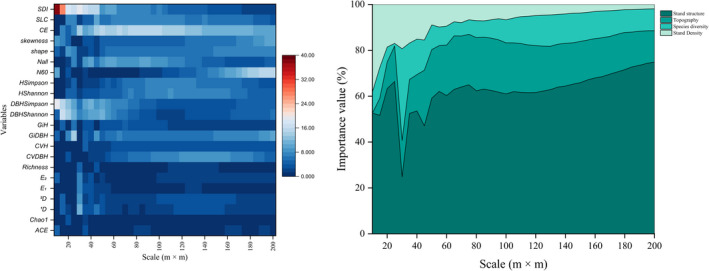
Left: Map depicting the relative importance values of variables patterns. The color from blue to red means the observed values are from low to high. Right: The relative importance value of the explanatory variables' categories at each scale

**FIGURE 6:**
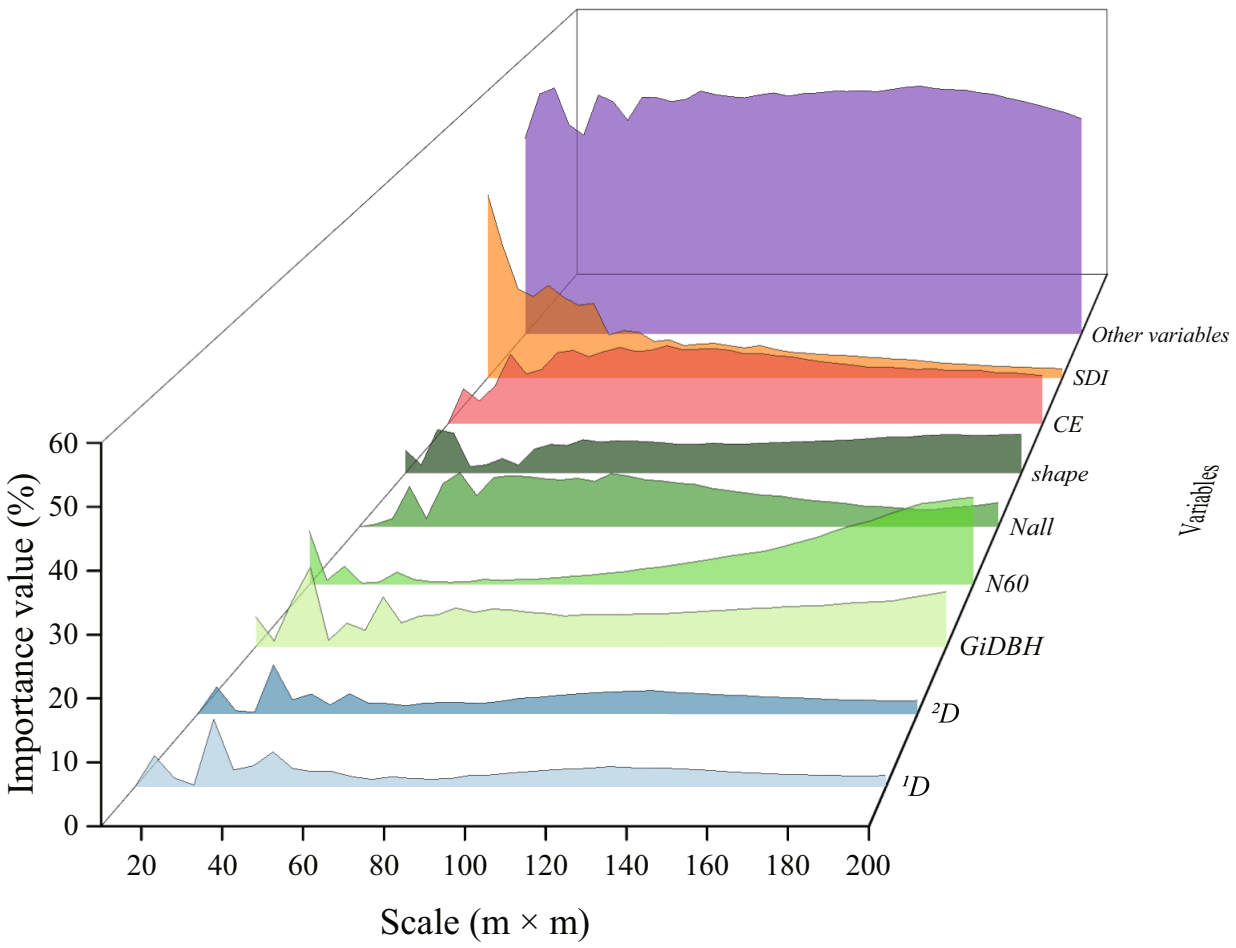
Map depicting the relative importance value patterns. The different color systems represent the different variables' categories

The relative importance value of the explanatory variables changed with scale. The importance of structural predictors tends to increase with increasing scale (Figure 5). Of structural predictors, stand structural diversity had the strongest effect sizes in general at the scale of 10–50 m and 110–115 m, while stem density of all trees was the best factor at the scale of 55–105 m, and at the scale of 120–155 m, tree size inequality was the most dominant variable, and at the scale of 160–200 m, stem density of big trees showed superiority (Figure 5). The importance of topographic factors at medium scale was better than that on small and large scale, and the performance of *CE* was stronger constantly than that of *SLC* (Figure 5). Of species diversity variables, ^*1*^ *D* and ^*2*^ *D* were the excellent predictors across all grain sizes (Figure 5), though their importance values were lower than 5% (except for 30 m) (Figure 6). SDI dominated increasingly with decreasing scales (Figure 6).

## 4 DISCUSSION

This study uses repetitive measurements of a 30‐ha broadleaved Korean pine forest in Jiaohe Forestry Experimental Administration Bureau of Jilin Province, China to estimate forest productivity for a five‐year period. We analyzed differences in the contribution of explanatory variables at different area scales, and the variation in the goodness of model fit. Our results highlight the fundamental role of scale in determining the relationship among four factors (topography, diversity, structure, and density) and biomass productivity per unit area and time. Lin et al. (2013) and Xu et al. (2015) captured biomass spatial variances in 24‐ha and 25‐ha forests, respectively. Therefore, we believe our results are credible in such 30‐ha forest. We will discuss the accuracy of the RF models at different scales, and the scale effect regarding the associations between the explanatory variables and forest productivity.

### 4.1 Accuracy of the RF models at different scales

This study shows that the estimates of biomass productivity are affected by plot size. The accuracy of the RF model in estimating biomass productivity increases with increasing scale. Lin et al. (2013) and Keller et al. (2001) have shown that the variability of AGB decreased with increasing quadrat size. Similar results were reported by Sullivan et al. (2018) in tropical rainforests in South America and Asia, and by Chave et al. (2004) in a rainforest in Panama, plus by Rodriguez‐Hernandez et al. (2021) in a subtropical forest in China. Our study agrees with these findings with comparable results.

A small scale would provide less confident information and capture less characteristics in contrast to a big one (Rodriguez‐Hernandez et al., 2021). This may be because too few samples may produce unreliable results (Leao et al., 2021), which also coincides with the statement that the bigger the number of sampling units, the greater the likelihood that new samples will lead to the same response (Brooks & Barcikowski, 2012). The spatial heterogeneity of sampling units can cause difficulties for community surveys, but large sampling areas are fundamental for reflecting community characteristics. Indeed, determining the optimum sampling area depends on the manpower and material resources consumed by the field survey and the accuracy required by the investigators. Hetzer et al. (2020) showed that 1 ha is the effective area for mean biomass estimation with sufficient precision in South America (Hetzer et al., 2020). We consider a sampling area of at least 140 × 140 m to reach the requirements for estimates of biomass productivity in this region (Figure 4 and Figure A.3), as the turning point in *R* ^2^ and rRMSE indicates the minimal scale for effective sampling is the above scale, sampling at a median scale may be more cost effective (*R* ^2^ > 0.7, rRMSE <20%). Using 3‐PGmix model, Xie, Lei, and Shi (2020); Xie, Wang, and Lei (2020) explored the impacts of climate change on the biological rotation of *Larix olgensis* plantations for timber production and carbon storage in 492 sample plots of 0.0667 ha each in northeast China. Based on the results of this study, the results of these previous research may have some problems. Admittedly, a larger sampling area will lead to higher accuracy, but at the same time undoubtedly increase the manpower, material, financial resources consumed. Thus, sampling design is often faced with a dilemma (Peck & Zenner, 2021) that the proper sampling plot scale to assess forest productivity must take productivity variability and spatial distribution into consideration; however, it is difficult to predict such variables before an inventory is conducted. Hence, our results provide potential guidance for future sampling schemes.

### 4.2 Scale dependence of the association between explanatory variables and AGB productivity

Our results indicated the most critical structure predictors were different at different scales. At the scale of 10–50 m and 110–155 m, DBH diversity and tree size inequality had significant links to productivity not only because multilayered forest structure can capture light and other resources easier (Ali et al., 2016; Yachi & Loreau, 2007), but also because tree size inequality plays a vital role in interfering the indirect impacts on biodiversity and abiotic factors on forest AGB productivity (Rodriguez‐Hernandez et al., 2021). At the scale of 55–105 m and 160–200 m, stem density were the best factors, which is in line with Rodriguez‐Hernandez et al. (2021). The importance of large trees increased with the increasing scale, which is different from the results of Rodriguez‐Hernandez et al. (2021). This can be explained that we studied the net biomass change, not the biomass. It is demonstrated that large trees have limited contribution to annual biomass production in an old‐growth forest (Ligot et al., 2018). Similar to Rodriguez‐Hernandez et al. (2021), *CE* had the greatest importance values at a large scale due to the various spatial distribution of topographic conditions. *CE* is the combination of aspect and elevation, which reflects complex moisture‐fertility effects (Xiang et al., 2016). The cosine of aspect gets its maximum value at north and it gets its minimum value at south. Furthermore, the natural logarithm of elevation increases monotonically. High importance values of *CE* demonstrated north aspects at high elevations or south aspects at low elevation had the biggest influence on biomass productivity at a large area scale. Species diversity were less important variable to project productivity across all scales in line with Sullivan et al. (2017), Fotis et al. (2018), Hao et al. (2018), Ali et al. (2019a), and Rodriguez‐Hernandez et al. (2021), which might be related to competitive exclusion (Ali et al., 2016; Grace et al., 2016). However, our study only pay attention to taxonomic diversity, which is just one of the facets of diversity. Since we established virtual sample plots, the other two diversity facets (i.e., functional diversity and phylogenetic diversity) are difficult to incorporate in our study. In that case, we would need to sample all the trees in the 30‐ha field plot, which is obviously difficult to achieve. But we still hope that in the future we can achieve such work, as this will make our study more comprehensive. There exists no single correct scale which forest ecosystems AGB productivity should be studied under the premise of suitable accuracy. That means readers can select the variables, which play important role in forest AGB productivity based on their intentions from Figure 5.

## 5 CONCLUSIONS

Our study shows that the estimation accuracy of AGB productivity is affected by scale. As the area scale increases, a greater number of factors contribute to the accuracy of productivity estimates. This important result could only be achieved based on a large fully enumerated and remeasured field plot. The results are essential for setting minimum sampling areas required for estimating AGB productivity in broadleaved Korean pine forests in Northeastern China, and possibly elsewhere. Further research is required to explore the optimum balance between sampling area, and assessment cost.

## AUTHOR CONTRIBUTIONS

**Jingyuan He:** Formal analysis (lead); methodology (lead); writing – original draft (lead). **Chunyu Fan:** Writing – original draft (supporting). **Yan Geng:** Formal analysis (supporting); funding acquisition (equal); writing – original draft (supporting). **Chunyu Zhang:** Funding acquisition (equal). **Xiuhai Zhao:** Conceptualization (lead); data curation (lead); methodology (supporting); supervision (lead). **Klaus von Gadow:** Writing – original draft (supporting).

## CONFLICT OF INTEREST

The authors declare that they have no known competing financial interests or personal relationships that could have appeared to influence the work reported in this paper.

## Supporting information


**Appendix S1** Supporting Information

## Data Availability

The data that support the findings of this study can be accessed on Figshare: https://doi.org/10.6084/m9.figshare.20151827.
