# Supplementary material for: Assessing scale‐dependent effects on Forest biomass productivity based on machine learning
Source: Ecol Evol. 2022 Jul 13;12(7):e9110. doi: 10.1002/ece3.9110 (PMC9277413; doi:10.1002/ece3.9110)
Supplement: Supplementary file 1 — Appendix S1 Supporting Information [file ECE3-12-e9110-s001.docx]

**Table A.1** Species information in the permanent forest plot. Based on the Engler classification system.

| No. | Species | Genues | Family |
| --- | --- | --- | --- |
| 1 | *Betula costata* | *Betula* | Betulaceae |
| 2 | *Betula platyphylla* | *Betula* | Betulaceae |
| 3 | *Betula dahurica* | *Betula* | Betulaceae |
| 4 | *Carpinus cordata* | *Carpinus* | Betulaceae |
| 5 | *Maackia amurensis* | *Maackia* | Fabaceae |
| 6 | *Quercus mongolica* | *Quercus* | Fagaceae |
| 7 | *Juglans mandshurica* | *Juglans* | Juglandaceae |
| 8 | *Tilia amurensis* | *Tilia* | Malvaceae |
| 9 | *Tilia* *mandshurica* | *Tilia* | Malvaceae |
| 10 | *Fraxinus mandchurica* | *Fraxinus* | Oleaceae |
| 11 | *Fraxinus chinensis* subsp. *rhynchophylla* | *Fraxinus* | Oleaceae |
| 12 | *Syringa reticulata* subsp. *amurensis* | *Syringe* | Oleaceae |
| 13 | *Abies holophylla* | *Abies* | Pinaceae |
| 14 | *Abies nephrolepis* | *Abies* | Pinaceae |
| 15 | *Pinus koraiensis* | *Pinus* | Pinaceae |
| 16 | *Prunus maximowiczii* | *Prunus* | Rosaceae |
| 17 | *Malus baccata* | *Malus* | Rosaceae |
| 18 | *Padus avium* | *Padus* | Rosaceae |
| 19 | *Sorbus alnifolia* | *Sorbus* | Rosaceae |
| 20 | *Phellodendron amurense* | *Phellodendron* | Rutaceae |
| 21 | *Populous davidiana* | *Populous* | Salicaceae |
| 22 | *Populus koreana* | *Populous* | Salicaceae |
| 23 | *Salix koreensis* | *Salix* | Salicaceae |
| 24 | *Acer mono* | *Acer* | Sapindaceae |
| 25 | *Acer* *mandshuricum* | *Acer* | Sapindaceae |
| 26 | *Acer tegmentosum* | *Acer* | Sapindaceae |
| 27 | *Acer ukurunduense* | *Acer* | Sapindaceae |
| 28 | *Acer triflorum* | *Acer* | Sapindaceae |
| 29 | *Ulmus davidiana* var. *japonica* | *Ulmus* | Ulmaceae |
| 30 | *Ulmus macrocarpa* | *Ulmus* | Ulmaceae |
| 31 | *Ulmus laciniata* | *Ulmus* | Ulmaceae |

**Table A.2** Equations for the calculation of above-ground biomass for each tree species. AGB represents the stem above-ground biomass (in g); dbh refers to the diameters at breast height (in cm).

| No. | Equations | Species involved |
| --- | --- | --- |
| 1 | AGB=0.090*(dbh*10)^2.696 | *Padus avium* |
| 2 | AGB=0.395*(dbh*10)^2.300 | *Syringa reticulata* subsp. *amurensis* |
| 3 | AGB=0.527*(dbh*10)^2.217 | *Acer mandshuricum*  *Acer tegmentosum*  *Acer ukurunduense*  *Acer triflorum* |
| 4 | AGB=10^(1.606+2.668*log10(dbh)) | *Tilia amurensis*  *Tilia mandshurica* |
| 5 | AGB=10^(1.826+2.558*log10(dbh)) | *Salix koreensis*  *Betula dahurica*  *Malus baccata*  *Populous davidiana*  *Populus koreana*  *Sorbus alnifolia*  *Prunus maximowiczii* |
| 6 | AGB=10^(1.930+2.535*log10(dbh)) | *Acer mono* |
| 7 | AGB=10^(1.942+2.232*log10(dbh)) | *Phellodendron amurense* |
| 8 | AGB=10^(2.002+2.456*log10(dbh)) | *Quercus mongolica* |
| 9 | AGB=10^(2.159+2.367* log10(dbh)) | *Betula platyphylla* |
| 10 | AGB=10^(2.213+2.417*log10(dbh)) | *Fraxinus chinensis* subsp. *rhynchophylla* |
| 11 | AGB=10^(2.214+2.400*log10(dbh)) | *Betula costata* |
| 12 | AGB=10^(2.216+2.408*log10(dbh)) | *Fraxinus mandchurica* |
| 13 | AGB=10^(2.235+2.287*log10(dbh)) | *Juglans mandshurica* |
| 14 | AGB=10^(2.236+2.144*log10(dbh)) | *Pinus koraiensis* |
| 15 | AGB=1000*0.0737*(dbh)^2.51264 | *Abies nephrolepis*  *Abies holophylla*  *Maackia amurensis* |
| 16 | AGB=1000*0.09802*(dbh)^2.2993 | *Ulmus davidiana var. japonica*  *Ulmus macrocarpa*  *Ulmus laciniata*  *Carpinus cordata* |

**Table A.3** Definition, units for the key forest attributes studied here.

| Category | Variable | Definition | Unit |
| --- | --- | --- | --- |
| AGB Productivity | $P_{ij}$ | Forest productivity measured in periodic annual increment in terms of the $ij$th quadrat net biomass change | Mg·ha^-1^·year^-1^ |
| Topography | ${CE}_{ij}$ | Cosine of aspect interacted with the natural logarithm of elevation of the $ij$th quadrat | Unitless |
|  | $\mathrm{SL}C_{ij}$ | Tangent of slope interacted with cosine of aspect of the $ij$th quadrat | Unitless |
| Species diversity | $Richness_{ij}$ | Tree species richness, the number of live tree species observed in the $ij$th quadrat | Unitless |
|  | ${{}^{1}D}_{ij}$ | Hill number of Order 1, the exponential form of Shannon entropy of the $ij$th quadrat | Unitless |
|  | ${{}^{2}D}_{ij}$ | Hill number of Order 2, the inverse of  Gini‐Simpson index of the $ij$th quadrat | Unitless |
|  | $E_{1_{ij}}$ | The exponential form of Shannon entropy divided by species richness in the $ij$th quadrat | Unitless |
|  | $E_{2_{ij}}$ | The inverse of Gini‐Simpson index divided by species richness in the $ij$th quadrat | Unitless |
|  | $ACE_{ij}$ | An elaboration of an earlier estimator, based on the ratio between rare and common species in the $ij$th quadrat | Unitless |
|  | $Chao1_{ij}$ | The ratio between the singletons and the doubletons in a given sample in the $ij$th quadrat | Unitless |
| Stand structure | ${N60}_{ij}$ | Number of large trees in the $ij$th quadrat | Unitless |
|  | $GiDBH_{ij}$ | Gini index of diameter at 1.3m in the $ij$th quadrat | Unitless |
|  | $GiH_{ij}$ | Gini index of tree height in the $ij$th quadrat | Unitless |
|  | $CVDBH_{ij}$ | Coefficient of variation of diameter at 1.3m in the $ij$th quadrat | Unitless |
|  | $CVH_{ij}$ | Coefficient of variation of tree height in the $ij$th quadrat | Unitless |
|  | $DBHShannon_{ij}$ | Shannon index of diameter at 1.3m in the $ij$th quadrat | Unitless |
|  | $DBHSimpson_{ij}$ | Simpson index of diameter at 1.3m in the $ij$th quadrat | Unitless |
|  | $HShannon_{ij}$ | Shannon index of tree height in the $ij$th quadrat | Unitless |
|  | $HSimpson_{ij}$ | Simpson index of tree height in the $ij$th quadrat | Unitless |
|  | ${Nall}_{ij}$ | Number of stems in the $ij$th quadrat | Unitless |
|  | ${shape}_{ij}$ | The shape parameter of the Weibull distribution fitted to all individuals’ DBH data in the $ij$th quadrat | Unitless |
|  | ${skewness}_{ij}$ | Skewness of the log-normal distribution fitted to all individuals’ DBH data in the $ij$th quadrat | Unitless |
| Stand density | $SDI_{ij}$ | The number of trees per unit area of stand with standard average diameter in the $ij$th quadrat | Unitless |


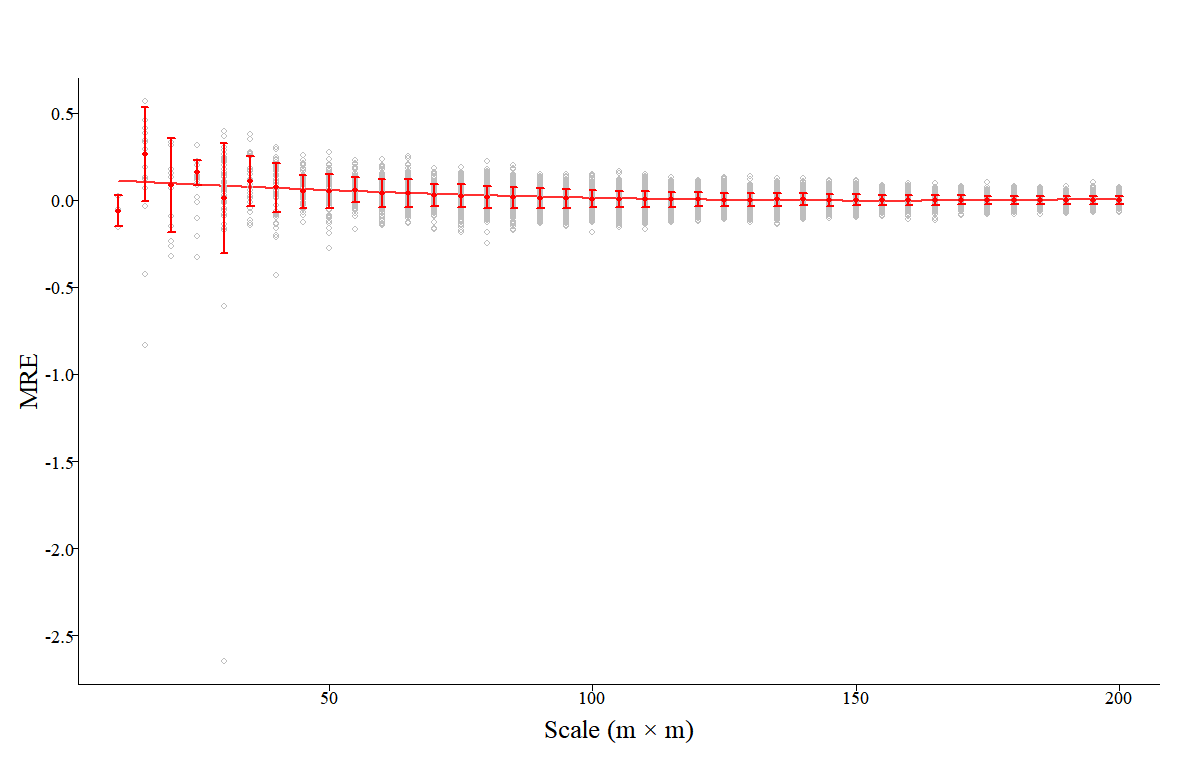


**FIGURE A.1** Boxplot of the mean relative error (MRE) changes modeled by the RF algorithm at each quadrat area scale. The solid line represents the mean trend line values, whereas dots with horizontal bars represent mean the MRE for each quadrat size value and its standard deviation (SD).


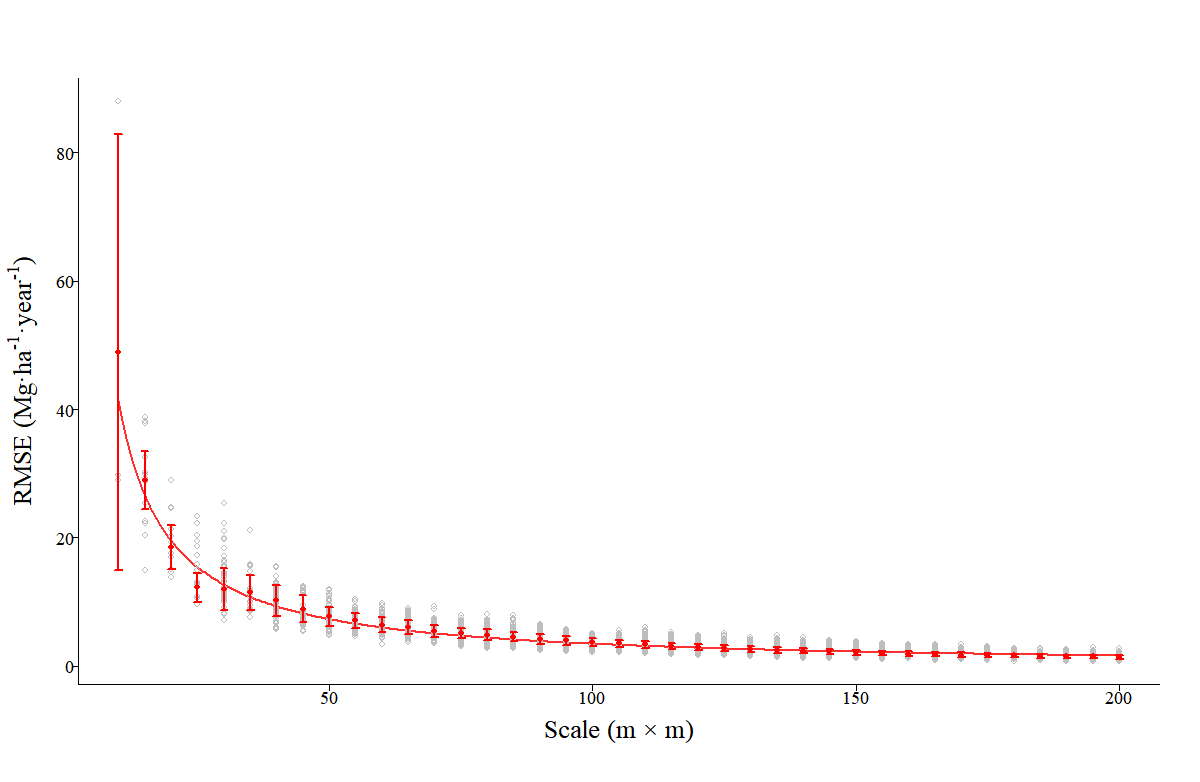


**FIGURE A.2** Boxplot of the root-mean-squared error (RMSE) changes modeled by the RF algorithm at each quadrat area scale. The solid line represents the mean trend line values, whereas dots with horizontal bars represent mean the RMSE for each quadrat size value and its standard deviation (SD).


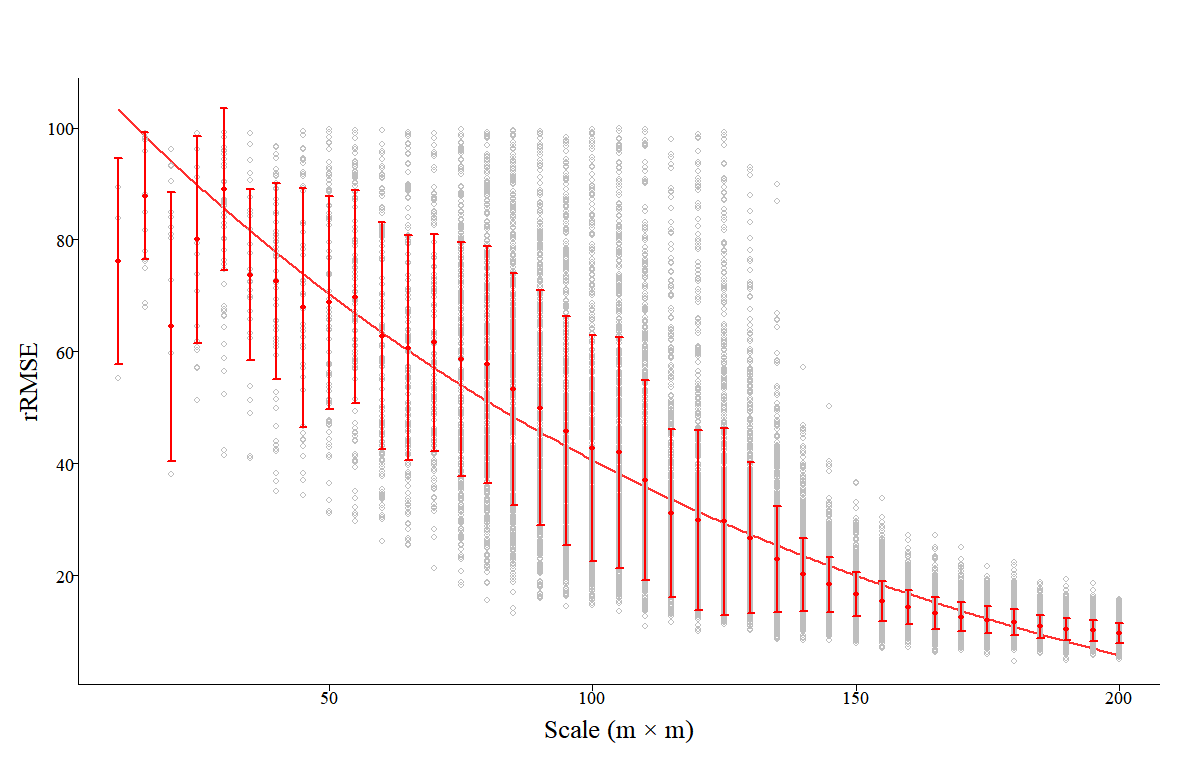


**FIGURE A.3** Boxplot of the relative root-mean-squared error (rRMSE) changes modeled by the RF algorithm at each quadrat area scale. The solid line represents the mean trend line values, whereas dots with horizontal bars represent mean the rRMSE for each quadrat size value and its standard deviation (SD).
